# How nurses face a new era of genomics medicine and precision health: Oncology nurse clinicians’ perspective

**DOI:** 10.1016/j.apjon.2024.100506

**Published:** 2024-05-11

**Authors:** Suzanne So-Shan Mak, Martin Leong-Tat Chan

**Affiliations:** Department of Clinical Oncology, New Territories East Cluster of Hospital Authority, Hong Kong SAR, China; Department of Clinical Oncology, Prince of Wales Hospital, Hong Kong SAR, China

Genetics and genomics is a newly emerging field in biochemistry, molecular biology, and health care. In health care, genetics has typically focused on variations in a single gene when determining the cause of a health condition. Genomics is the study of an organism's complete set of genetic information, including both the genes that code for proteins and the noncoding regions. The application of genomics is a breakthrough in health care in terms of pathogenic surveillance, provision of medical care, and food security.

In oncology, genetics and genomics is having a profound impact on precision medicine. Precision medicine describes a standard for healthcare delivery focusing on patient's clinical needs and characteristics, digital health, omics, and other biomedical technologies, data sharing, and data science to be clinically effective and successful.^1^ As clinical practice moves toward greater precision due to its better efficacy in controlling cancer, genomic technology plays an important role in the drastic advancement in cancer prevention and screening, diagnosis, treatment, and research.

In fact, the World Health Organization makes an advocacy for the application of genomics regarding promotion, implementation, collaboration, and attention to the ethical, legal, and social issues.^2^ These advocacies deserve attention in oncology as genetics, and genomics is a fast-developing subspecialty in oncology and is gaining growing importance and applicability. Oncology nurse also has an inevitable role and responsibilities in this emerging health process. However, to date, there is no clearly defined role of oncology nurses in genetics and genomic care. Therefore, it is time to have a discussion and deliberation regarding the scope of practice of oncology nurse in genetics and genomics.

Nursing metapradigm outlines the conceptual foundation of nursing and nursing practice, which constitutes the philosophy, attitudes, and scope of scientific studies of the nursing profession. By revisiting the nursing metapradigm, oncology nurses could gain a better insight into the conceptual ideologies and basis of oncology nursing practice, which facilitates the discussion regarding the new emerging oncology nursing role in genetics and genomic care (Fig. 1).

**Fig. 1:**
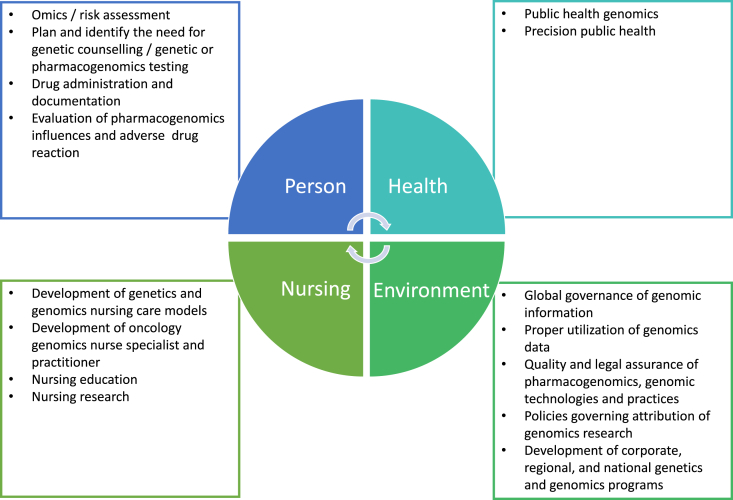
Emerging expanded oncology nursing roles in genetic and genomics care.

## Nursing metaparadigm and implications to genetics and genomics in oncology nursing

### Fawcett's nursing metaparadigm

Fawcett is one of the nursing metaparadigms commonly adopted in describing nursing practice. Under the metaparadigm, nursing would interact with person, environment, and health and develop three specific relationships.^3^ They are person–health, person–health–environment, and person–health–nursing relationships. A person in Fawcett's metaparadigm is defined as an individual in a definite family, culture, and society and is characterized by the composition of physical, psychological, social, and spiritual dimensions. Health is regarded as the continuum from birth to death. Environment refers to all cultural, sociopolitical, and socioeconomic conditions that entail personal or populational health. Nursing is “the study of human health and illness processes. Nursing practice is facilitating, supporting, and assisting individuals, families, communities, and /or societies to enhance, maintain, and recover health and to reduce and ameliorate the effects of illness. Nursing's relational practice and science are directed toward the explicit outcome of health-related quality of life within the immediate and larger environmental context” (p. 1265).^4^

## Person perspective

The appreciation and respect of a person's bodily condition as well as personal meanings and perception are where nursing is distinctive from other biomedical, psychosocial, or spiritual professionals. These unique characteristics support nursing capable enough to provide person-centered holistic care in the context of complex ethical dilemmas and psychosocial impacts arising from genetic and genomics issues. In fact, an oncology nurse should be equipped with basic knowledge and core competence in genetics and genomics. Incorporating to their current knowledge and skills in both nursing and oncology, an oncology nurse could be helpful in at least four major areas in genetic and genomics oncological care.

a. Omics/risk assessment

Embedding genomics and pharmacogenomics into nursing practice can be achieved by enhancing the assessment during the nursing process. The omics/risk assessment^5^ includes assessing family history whether patients or their family have had a problem metabolizing drugs; identifying patient's ancestry and ethnicity as ethnically related diseases are associated with ethnic groups; and establishing the probability of genetic condition or predisposition to an adverse drug reaction, which thus helps to improve medication selection, and tailor and optimize the management of symptoms. In the nursing area, an accurate family health history must be collected by nurses as this represents a key nursing action that can help identify the need for omics testing for various risk factors and disease conditions.

b. Plan and identify the need for genetic counseling/genetic or pharmacogenomics testing

Nurses can work with multidisciplinary teams to identify patients who could benefit from genomic testing; discuss testing with patients; use biomarkers to objectively identify health risks and identify more precisely the various mechanisms responsible for adverse clinical conditions, identify patients who would benefit from a referral to clinical genetic services, and, in this way, nurses can effectively develop, target, and finalize personalized care or interventions to improve the management of patients.

c. Drug administration and documentation

The nursing profession has license requirements and professional standards for nursing medication administration and documentation. The previous standards charged nurses with the five rights: the right patient, right dose, right drug, right route, and right time. Today, with the foundation of pharmacogenomics in precision health in diverse ethnic populations, it is the right drug, for the right person, at the right dose regardless of age.^6^ Most medications that the patients were taking were associated with pharmacogenomic information. Pharmacogenomics combines the science of drugs and their metabolism, with the genetics of enzymes that metabolize drugs to develop effective medications, safe medications, and doses tailored to the person's genetic profile. Nursing roles are pivotal in supporting the precision delivery of medications according to modern concepts of pharmacogenetics. In this way, nurses may involve patients and families in health education, also on the meaning and the utility of omics tests, thus supporting adequate care delivery.

d. Evaluation of pharmacogenomic influences and adverse drug reaction

Some drugs are linked to adverse reactions because of variations in the human genome, specifically, DNA sequence variants, being able to affect a drug's pharmacokinetics, pharmacodynamics, efficacy, and safety. Medications are broken down in the liver by enzymes that may be affected by genomics. Taking warfarin as an example; it is used to prevent clotting in persons with arrhythmias, deep vein thrombus, after coronary surgery, and extensive orthopedic surgery. The CYP2C9, VKORC1, and CYP4F2 are genes known to be associated with responses leading to excessive bleeding in patients.^7^ These may result in the nurse observing epistaxis, hematuria, and bleeding symptoms in patients. Nursing assessments, observations and documentation are critical in determining if adverse drug reactions are occurring. Pharmacogenomics is an important factor in precision health translated to nursing documentation of medication administration and observation of adverse reactions.

## Health perspective

Nursing has a philosophical orientation of health as a continuum instead of a single or series of fragmented episodes of events of bodily condition. Illness prevention and health promotion are also the scope of practice of nursing; therefore, role expansion in oncology nursing not only focuses on secondary and tertiary care, but oncological genetic and genomic care should also be incorporated in public health and primary care nursing. Indeed, public health genomics is the practice of precision public health that incorporates epidemiology and genomic information and techniques to inform public health policy and health service development.^8^ Nursing could expand its roles in public health genomics in terms of genomic screening, genetic counselling, and public health policy advocacy.

## Environment perspective

Fawcett viewed nursing as a social mandate in supporting the healthcare system in which inevitably has the social and political responsibility in the notion of advocating social health perception and health-related quality of life.^4^ Apart from that, a person interacts and is codependent with the environment. Nursing should take personal values and perceptions into the context of social and political realities. As such, an oncology nurse acknowledges those social constraints that jeopardize personal health, life possibilities, and health service access and advocates for population health equity.

In terms of genetic and genomics services, this is very consistent with the World Health Organization's advocacy in dealing with the ethical and social ramifications of genomics, such as global governance of genomic information, proper utilization of genomics data, quality and legal assurance of pharmacogenomics, genomic technologies and practices, as well as policies governing the attribution of genomics research.^2^ Moreover, nurses should actively participate in building sustainable and cost-effective corporate, regional, and national genetic and genomic programs in terms of infrastructure procurement and management as well as personnel training.

## Nursing perspective

Nursing is the largest healthcare profession, and nurses practice in all healthcare settings. Therefore, nurses must be at the forefront of the integration of genetics and genomics into clinical practice. Based on Fawcett's nursing metaparadigm, professional nurses should perform their practice and provide nursing interventions based on own body of knowledge as well as focus on caring.^4^ To meet the expanded role of genetics and genomic oncology nursing, there is an urge for structural education to both basic and specialist training. Feasible access to genomic experts, support networks, infrastructure, and leadership would also share equal importance.^9^ Indeed, nursing leaders could take this opportunity to influence and utilize resources in developing new nursing specialist and practitioner positions and new care models to echo the pressing needs of genetics and genomics in oncology care. Nurse researchers are important companions in the integration of genetic and genomics applications in oncology nursing. Basic science research, translational research in genomic care model, toxicities management arising from novel precision therapies, health-related quality-of-life issues, as well as big data research are some of the core elements that deserve attention in strengthening the clinical development in genetic and genomics nursing.

## Ethics statement

Not required.

## Funding

This study received no external funding.

## CRediT authorship contribution statement

Mak SSS and Chan MLT conceived of the presented idea and wrote the article.

## Declaration of competing interest

The authors declare no conflict of interest. The corresponding author, Suzanne So-Shan MAK, serves as a member of the editorial board of the *Asia-Pacific Journal of Oncology Nursing*. The article has undergone the journal's standard publication procedures.

## Declaration of generative AI and AI-assisted technologies in the writing process

No AI tools/services were used during the preparation of this work.
